# Improper handling of vomitus as a risk factor in the human norovirus outbreak in a kindergarten in Wuyi County, Zhejiang Province, China

**DOI:** 10.1017/S0950268822000826

**Published:** 2022-05-17

**Authors:** Wanwan Sun, Zhifeng Pang, Yuanyuan He, Yijuan Chen, Jinren Pan, Jian Gao, Ziping Miao

**Affiliations:** 1 Zhejiang Provincial Center for Disease Control and Prevention, Hangzhou, China; 2 Jinhua Municipal Center for Disease Control and Prevention, Jinhua, Zhejiang, China; 3 Wuyi County Centre for Disease Control and Prevention, Jinhua, China

**Keywords:** Disinfection, human norovirus, kindergarten, outbreak, person-to-person

## Abstract

This study investigated an outbreak in a kindergarten in Wuyi County of acute gastroenteritis concerning a large number of students and teachers. We performed a case-control study, and collected information on the layout of the school, symptoms, onset time of all cases and vomiting sites. A total of 62 individuals fit the definition of probable cases; among these, there were 19 cases of laboratory-confirmed norovirus infection. Nausea and vomiting were the most common symptoms in the outbreak. Seven student norovirus patients vomited in the school. The odds ratio (OR) of norovirus illness was 15.75 times higher among teachers who handled or interacted with student vomitus without respiratory protection than compared to those without this type of exposure (OR 15.75, 95% CI 1.75–141.40). Nine samples were successfully genotyped; eight samples were norovirus GII.2[P16], one sample was norovirus GII.4 Sydney[P16]. This study revealed that improper handling of vomitus is a risk factor of norovirus infection. Therefore, more attention should be given to train school staff in knowledge of disinfection.

Human noroviruses, positive-sense single-stranded ribonucleic acid (RNA) viruses belonging to the family *Caliciviridae*, have become one of the major causes of gastroenteritis worldwide [1–3]. Noroviruses are categorised into five genogroups (GI–V), with GI, GII and GIV having the ability to cause infection in humans [4]. Noroviruses can be transmitted by the faecal–oral route or by inhalation of aerosol-containing virus particles from patient's vomitus [5, 6]. Noroviruses are highly contagious; it can rapidly infect a person with only 18 viral particles [7]. The common incubation period is 12–48 h, and typical symptoms after infection are as follows: diarrhoea, nausea, vomiting, abdominal pain and fever. Cases of norovirus infection are generally self-limited and mostly mild, with one or several symptoms usually lasting for 1–3 days [4, 8]. There are not currently any effective vaccines publicly available for norovirus.

A majority of norovirus cases and outbreaks occur in autumn and winter when the weather becomes cold. Outbreaks often occur in various types of schools, offices, nursing homes, hospitals and other places where people congregate [9–11]. The latent infection rate of norovirus in the general population is relatively high. A systematic review of scientific literature reported the pooled prevalence of norovirus in 187 336 patients with acute gastroenteritis was 18%, demonstrating that norovirus has become an important cause for cases of acute gastroenteritis [12]. Children with poor nutrition are particularly at risk of norovirus infection and even death. For example, children of Aboriginal and Torres Strait Islander populations in northern Australia have increased rates of malnutrition and electrolyte disturbance, and longer hospital stays following norovirus infection than children with adequate nutrition and clean food [13].

Norovirus has a unique mode of epidemic [14]. Among the 556 outbreaks reported between October 2016 and September 2018 in mainland China, 50.4% occurred in child centres, 27.9% in primary schools, 11.0% in middle schools, 4.3% in universities and 6.4% in other places (hospital, hotel, party, etc.); in terms of the route of transmission, among the 452 outbreaks identified, person-to-person was the predominant route (95.1%), followed by foodborne transmission (2.9%), waterborne (1.3%) transmission and 0.7% other [15]. Among the 661 norovirus outbreaks in Beijing from 2014 to 2017, 3.8% were caused by GI genogroup norovirus, 95.6% by GII genogroup norovirus and 0.6% by both GI and GII [16].

Zhejiang is one of the provinces with a large number of reported norovirus outbreaks from 2014 to 2017 [1, 17]. On 11 December 2020, the Center for Disease Control and Prevention of Zhejiang (ZJCDC) received a report of a large number of children from a kindergarten in Wuyi County with sudden vomiting and diarrhoea; however, the cause was not very clear. To control and find the cause of the outbreak, we conducted an epidemiological investigation and laboratory testing to identify the causative agent and provide guidance on effective control measures for similar future outbreaks.

## Methods

### Case definition and search

A clinical case was defined as follows: students and staff of the kindergarten who had diarrhoea more than three times within 24 h, with the character of stool changing (thin watery stool), or had vomited more than two times within 24 h since 1 December 2020. A laboratory-confirmed case was defined as an anal swab, stool or vomitus sample from a clinical case that tested positive for norovirus by real-time reverse transcription polymerase chain reaction (RT-PCR).

### Epidemiological investigation

Probable and laboratory-confirmed cases were investigated. The environment of the kindergarten, basic information on norovirus cases and the recent number of patients with gastrointestinal symptoms such as vomiting and diarrhoea were first collected. Secondly, students, teachers and other staff of the kindergarten were assessed with regard to clinical symptoms, activities, contacts, dietary items, beverage consumption and water supply.

As kindergarten students are relatively young and cannot cooperate well with an investigation, we chose teachers, logistics and management staff as the subjects to conduct a case-control study.

### Sample collection

Environmental samples were collected from the cafeteria kitchenware, food, classroom desktops, washrooms, teacher offices and hot-water storage tanks. Stool samples were obtained from the food handlers, students, teachers and other working staff with related gastrointestinal symptoms.

### Screening and sequencing for gastroenteritis pathogens

According to the technical procedures of diarrheal pathogenic spectrum surveillance formulated by China CDC [18], stool samples were collected from the children and staff with acute gastroenteritis at the kindergarten in Wuyi County. The samples were processed into 10% (w/v) suspensions in phosphate-buffered saline and centrifuged at 2000×*g* for 10 min. Viral RNA was extracted from 200 μl of the supernatant using a Qiagen RNeasy Mini kit (QIAGEN 74104) according to the manufacturer's instructions. The eluted RNA was stored at −80 °C until use. A commercial real-time PCR kit for norovirus GI and norovirus GII (Liferiver, China) was used. Positive samples were amplified by One Step RT-PCR Kit, TAKARA DRR055A, with norovirus primers JV12 [19] (ATACCACTATGATGCAGATTA) and G2SKR [20] (CCRCCNGCATRHCCRTTRTACAT), which amplify a partial region of ORF1 and a partial region of ORF2 using the following thermal cycling profile: 50 °C for 30 min; 94 °C for 2 min; and 35 cycles of 94 °C for 30 s, 51 °C for 30 s and 72 °C 1 min. Samples producing an approximately 900 bp product were sequenced with the Sanger method. All sequences were manually edited using DNAStar software and then classified using the Norovirus Genotyping tool [21].

### Statistical analysis

All epidemiologic and laboratory data were entered into Excel 2016, and analyses were performed using R 3.6.1 (the R project for statistical computing, Vienna, Austria). Trees were generated by using the Neighbour-Joining method with 1000 bootstrap replicates implemented in MEGA 10 (https://www.megasoftware.net). All statistical tests were two-sided, and *P* values <0.05 were considered statistically significant.

## Results

### Descriptive epidemiology

**Fig. 1.**
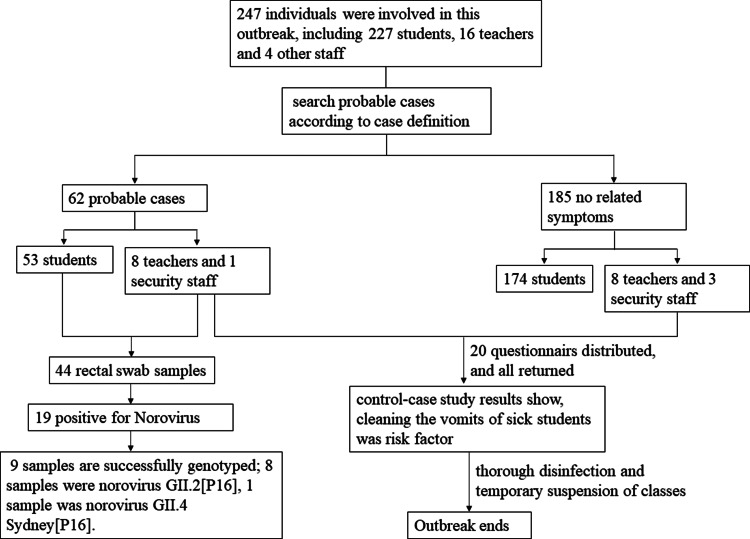
A brief flow-diagram of the Norovirus outbreak investigation in Wuyi, Zhejiang Province.

There are four grades in this kindergarten: baby, junior, middle and senior. Each grade has two classes, and each class has two teachers. The school has a total of 227 students, 16 teachers and 2 logistics and management staff members. This kindergarten has an independent cafeteria, which provides daily lunches for the teachers and students, and the food and water are the same for both. The whole investigation process of the outbreak in this study is shown in Figure 1.

In this outbreak, a total of 62 individuals fit the definition of the probable case; among them, there were 15 cases of laboratory-confirmed norovirus infection. Fifty-three of the 62 cases were students aged between 3 and 7 years old; eight cases were teachers, where one of the eight cases was the security guard aged 60.

There were 31 male and 31 female infected individuals, and 84 male and 101 female non-infected individuals, with no significant difference (*χ*^2^ = 0.39, *P* = 0.53). There was also no statistically significant difference between the cases and non-cases with regard to role, namely, student or teacher (*χ*^2^ = 3.08, *P* = 0.08). The odds ratios (ORs) of the top grade (*χ*^2^ = 4.24, *P* = 0.04) and staff (*χ*^2^ = 4.58, *P* = 0.03) were statistically significant (Table 1).

**Table 1.** Comparison of cases and non-cases in different groups

Categories	Cases	Non-cases	Total	*χ*^2^	*P*	OR	95% CI
Gender							
Male	31	84	115	0.39	0.53	1.20	0.68–2.14
Female	31	101	132				
Role							
Student	53	174	227	3.08	0.08	0.44	0.17–1.12
Staff	9	11	20				
Grades							
Senior	28	57	85	4.24	0.04	1.85	1.03–3.34
Middle	13	50	63	0.90	0.34	0.72	0.36–1.43
Junior	11	49	60	1.93	0.17	0.60	0.29–1.24
Baby	1	18	19	3.24	0.07	0.15	0.02–1.16
Staff	9	11	20	4.58	0.03	2.69	1.06–6.83

OR, odd ratio; CI, confidence interval.

### Clinical symptoms

Nausea (89.06%) was the most common symptom; 79.69% had vomiting, 68.75% had abdominal pain, 9.38% had diarrhoea and 6.75% had fever (Table 2). All cases in this outbreak were mild, and no deaths occurred (Table 2).

**Table 2.** Distribution of major symptoms

Symptom	No. of cases (*N* = 62)	Proportion (%)
Fever	17	27.42
Nausea	56	90.32
Abdominal pain	42	67.74
Vomit≥2/day	50	80.65
Diarrhea≥3/day	6	9.68

In this survey, we collected blood test results for 15 students who went to the hospital. Abnormal white blood cell and central granulocyte counts were both highest at 93.33%, though the red blood cell count and rapid CRP level of all 15 students were normal (Table 3).

**Table 3.** Blood test result for cases

Blood test items	Max	Min	Median	S.D.	Abnormal rate (%)
White blood cell count	24.60	5.40	20.30	4.80	93.33
Central granulocyte count	88.50	43.70	15.50	4.27	93.33
Absolute value of lymphocyte	6.50	1.20	3.60	1.51	53.33
Absolute value of monocytes	1.80	0.60	1.10	0.46	80.00
Absolute value of eosinophil	0.27	0.08	0.10	0.08	6.67
Basophilic absolute value	0.07	0.01	0.04	0.02	6.67
Red blood cell count	5.03	4.09	4.65	0.28	0.00
Haemoglobin	137.00	119.00	130.00	5.65	13.33
Platelet count	561.00	216.00	396.00	96.79	73.33
Rapid CRP	<0.50	<0.50	<0.50	–	0.00

S.D., standard deviation.

### Time distribution

The first two cases of this outbreak occurred on 10 December 2020 at 6:00–12:00 h. The third case occurred on the same day as the first case at 12:00–16:00 h. All three cases first developed gastrointestinal symptoms at school on 10 December, two of them vomited in the classroom and the corridor, and all three of them had symptoms of nausea and abdominal pain. Thereafter, cases were successive. On the morning of 11 December, five students and teachers of the kindergarten with similar clinical symptoms went to The First Hospital of Wuyi County, which is the nearest hospital to the school, attracting the attention of the hospital's management staff responsible for infectious disease, who then immediately reported it to the local public health department.

**Fig. 2.**
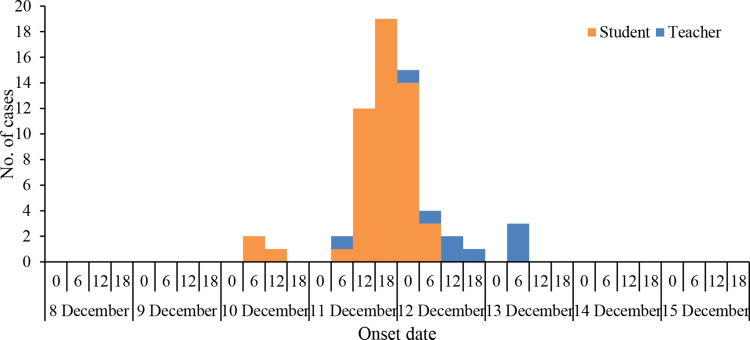
Date and time (hours) of symptom onset.

The number of cases peaked from 11 to 12 December. After the kindergarten was temporarily closed on 12 December, the number of cases declined sharply and decreased to three cases on 13 December. As a result, no new cases occurred after 14 December until the resumption of classes on 21 December (Fig. 2).

### Space distribution

**Fig. 3.**
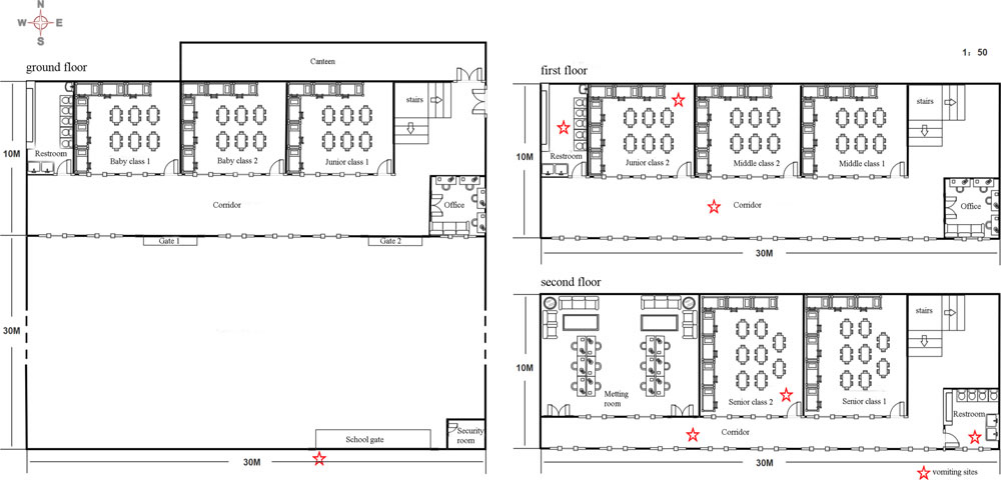
Probable locations of infection in the kindergarten outbreak.

The overall layout of the three floors of the kindergarten is shown in Figure 3. The school's eight classes are located on three floors of the teaching building. The ground floor contains two baby classes, one junior class and one teacher's office; the first floor contains two middle classes and one junior class; and the second floor contains two senior classes and teacher's offices. There were 62 cases on the three floors, including five on the ground floor, 26 on the first floor and 31 on the second floor.

The stars marked in Figure 3 indicate where the students vomited. Three students vomited on the first floor: junior class 2 (10 December), the corridor (10 December) and the restroom (11 December). Three students vomited on the second floor: senior class 2 (12 December), the corridor (11 December) and the restroom (11 December). Notably, a student vomited when he passed the school gate after school, and the school security guard helped him to deal with the vomitus and wipe his mouth on 11 December 2020.

The attack rate (AR) of senior class 1 was the highest at 42.22%, followed by junior class 2, middle class 2 and senior class 1, at 29.41%, 27.27% and 27.27%, respectively. Importantly, there was one positive case among the kindergarten director, two kitchen staff members and one security guard, with an AR of 25% (Table 4).

**Table 4.** The attack rate in different classes

Class	Floor	Cases		Total person	AR (%)
		Student	Teacher		
Senior class 1	Second floor	17	2	45	42.22
Senior class 2	Second floor	11	1	44	27.27
Middle class 1	First floor	5	2	34	20.59
Middle class 2	First floor	8	1	33	27.27
Junior class 1	First floor	9	1	34	29.41
Junior class 2	Ground floor	2	1	30	10.00
Baby class 1	Ground floor	0	0	10	0.00
Baby class 2	Ground floor	1	0	13	7.69
School logistics management personnel	Ground floor	0	1	4	25.00

### Pathogen detection

On 12 and 13 December, a total of 69 samples were collected, including 44 rectal swab samples from students, teachers and logistics staff. Twenty environmental swab samples were collected from classrooms, bathrooms, tableware or water buckets, and four food samples from the cafeteria. The positive rate of the laboratory specimens for teachers, students and logistics staff is 47.06%, 41.67% and 33.33%, respectively. Twenty-four environmental swab samples are tested as negative (Table 5).

**Table 5.** Laboratory test results for samples from the outbreak

Samples	No. of samples	Positive	Positivity rate (%)
Stool/rectal swab samples			
Teacher	17	8	47.06
Student	24	10	41.67
Logistics staff (security and cafeteria)	3	1	33.33
Environmental swab samples			
Classroom\washroom\tableware	12	0	0.00
Bucket	8	0	0.00
Food	4	0	0.00
Total	68	19	27.94

CI, confidence interval.

### Sequencing of laboratory specimens

Phylogenetic analysis of isolates from the outbreak revealed 19 positive samples of 44 rectal swab samples, which were sequenced in the laboratory of Zhejiang Provincial CDC. Among all norovirus GII-positive cases, nine were genotyped, in which one was genotyped as recombinant GII.4 Sydney[P16]; the other eight cases were classified as recombinant GII.2[P16] and one of the seven infected individuals carried an isolate with a mutation of GII. 2(P16).

**Fig. 4.**
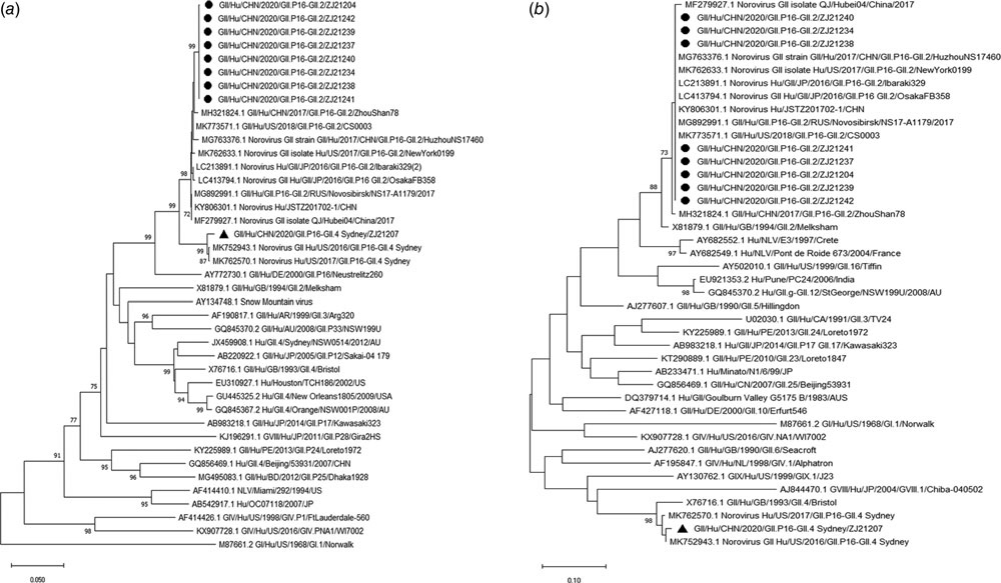
Phylogenetic trees of the norovirus GII partial-nucleotide sequences. (a) Analysis of the RNA-dependent RNA polymerase (RdRp) region; (b) analysis of the major capsid protein VP1 region.

Figure 4 displays the trees of the sequence results; Bootstrap values >70 are indicated at the nodes. Strains of sufficient nucleotide sequence length were included in the trees (GII.4 Sydney[P16] in this outbreak was labelled with black triangle and GII.2[P16] in this outbreak was labelled with black circle). Reference strains are shown with accession numbers.

### Case-control investigation

Staff had a statistically significant OR relative to the other classification groups. In this part, all staff including nine infected staff and 11 non-infected staff were interviewed. Table 6 summarises the ORs for each item. The odds of meeting the case definition were 15.75 times higher among teachers who handled or interacted with student vomit without respiratory protection compared to those without exposure or having used respiratory protection (OR 15.75, 95% CI 1.75–141.40). Compared with control participants, three risk factors, including ‘Have you been to the hospital to accompany students’, ‘Are you responsible for feeding students’ and ‘Number of student cases in your class’, showed no significant statistical difference.

**Table 6.** Case-control study for a norovirus outbreak among working staff in Jinhua City, China

Variables	Cases	Control	OR	*P* value	95% CI
Have ever handled student vomit without protective measures					
Yes	7	2	15.75	0.02	1.75–141.40
No	2	9			
Have you been to the hospital to accompany students					
Yes	6	2	9.00	0.07	1.14–71.04
No	3	9			
Are you responsible for feeding students					
Yes	7	6	2.92	0.37	0.41–20.90
No	2	5			
Number of student cases in your class					
>8	4	4	1.40	1.00	0.23–8.46
≤8	5	7			

AR, attack rate.

## Discussion

The results of this epidemiologic investigation indicated that, improper handling of norovirus vomitus was an important risk factor in the outbreak of norovirus in the kindergarten of Wuyi County, Zhejiang Province, China. The evidence is as follows: (1) the water and food consumed by all students, teachers and other staff were the same; (2) compared with teachers who never handled the students' vomitus, those who handled vomitus had a significant OR value for norovirus infection; and (3) the outbreak was quickly quelled after suspending classes temporarily and the implementation of thorough disinfection. The evidence above supports that improper handling of vomitus is a risk factor of interpersonal transmission of the virus.

Norovirus is increasingly recognised as an important pathogen of acute gastroenteritis. All age groups are vulnerable to infection, though mortality is low and tends to occur in those at age extremes [7]. Faecal–oral spread is the primary route of norovirus transmission, but humans can also be infected by close contact or contaminated food or water [22]. Although aerosol transmission comprises a relatively small proportion of transmission in reported studies, it often coexists with other modes of transmission, causing small-scale outbreaks.

Vomiting is one of the common symptoms of norovirus infection. Aerosol and object pollution formed by vomiting is a common pathway of infection [23, 24]. A patient can excrete the virus and infect other individuals during the incubation period of 2–5 days, generally during and following the acute phase. A patient can therefore remain infectious for 2–3 weeks [25, 26]. Makison Booth *et al*. found the existence of small droplets of norovirus fluid that travelled 3 m away from the vomiting site, and that the virus can survive being ejected even within small far-reaching droplets at concentrations capable of eliciting infection [27]. Evans *et al*. analysed an outbreak of viral gastroenteritis following environmental contamination at a concert hall, and found that children who sat on the same level of the auditorium as the index case who vomited on the same evening were much more likely to be ill than those seated elsewhere [28]. Alsved *et al*. have conducted an investigation to explore the association between symptoms of gastroenteritis and the presence of airborne norovirus, and to investigate the size of norovirus-carrying particles; they concluded their finding as recent vomiting is the major source of airborne norovirus and implied a connection between airborne norovirus and outbreaks. Additionally, the presence of norovirus RNA in submicrometre particles indicated that airborne transmission can be an important transmission route [29]. In this study, people were infected by inhaling air containing virus particles or touching the surface of contaminated objects when handling the vomitus with improper vomit-disposal measures, including not wearing appropriate protective masks, gloves and not using effective disinfectants.

The existence of recessive norovirus infection creates a prerequisite for the outbreak. Several cross-sectional surveys have examined the recessive infection rate of norovirus in children under 5 years of age, and the results showed that the percentage is quite different, with proportions in the UK [30] and Burkina Faso [31] exceeding 24%; however, research in China and Vietnam found a percentage of only 2.7–2.8% [32, 33]. A 2-year (October 2009–October 2011) follow-up study of children in a kindergarten in Brazil showed a rate of norovirus infection as high as 37.5% [34].

Norovirus genotypes vary with time, region and age group but the epidemic genotype did not vary much in China. A single genotype (GII) of norovirus has been dominant since the late 1990s [35, 36], and GII was also found to be the main dominant strain causing the norovirus-related outbreak in China. Miao *et al*. analysed norovirus outbreak surveillance data from 2016 to 2018 and found that 91.5% of the outbreaks were due to GII; with 5.5% due to GI, and that 2.5% were comprised of both GI- and GII-positive samples [15]. For the 94 norovirus outbreaks in China 2016, Wang *et al.* reported that GII.2 was the most common genotype (52%), followed by GII.3 (9%), multiple genotypes (9%), GII.4 Sydney 2012 (7%), GII.17 (5%), GII.6 (5%) and others (13%) [37].

Compared to norovirus outbreaks, which are mainly transmitted by contact and aerosols, water-borne and food-borne outbreaks are more likely to lead to a relatively larger range of population infections and outbreaks [38–40]. In fact, the correct treatment of vomitus and disinfection methods is of great significance for reducing second-generation cases [41]. Wang *et al*. designed a Vomit Disposal Kit for acute gastroenteritis caused by norovirus [42], which includes protective equipment for vomitus disposal, disinfection agents and disposal tools. The procedure for vomitus disposal is as follows: (1) evacuate people around the vomitus; (2) personnel should wear protective equipment, including a hat, two pairs of gloves, mask, shoe covers, etc., while disinfecting; (3) prepare a disinfectant solution with an effective chlorine concentration of 5–10 g/l [43], soak a towel in the bag with the previously mentioned disinfectant solution, cover the vomitus with the towel for 30–60 min; (4) pick up the towel covering the vomitus and place it into a yellow garbage bag for medical waste, and disinfect the gloves with 5% Iodophor disinfection cotton [44]; (5) remove the outer gloves and the protective equipment, and then remove the next pair of gloves and thoroughly clean the hands.

We took several control measures to stop the outbreak as soon as possible. First, temporarily stopping classes and avoiding student and teacher gatherings are important measures to control the spread of norovirus in schools that were infected. Second, morning health checks and illness-induced absences were implemented, with every student and staff member of the kindergarten reporting their health and the reason why they were absent from school. Third, all patients mandatorily stayed at home and returned to school 3 days after recovery. Infected teachers were sent to the CDC twice (one day apart) for rectal swab examinations before returning to work. Fourth, it is necessary to train teachers and students on norovirus prevention. The content of training should focus on how to correctly deal with probable contaminated vomitus, how to disinfect the environment that may be contaminated by the virus, and how to establish a student health report system for the norovirus epidemic season.

This study underscores the need for better handling of vomitus-containing noroviruses and proper disinfectant methods in response to the outbreak of norovirus acute gastroenteritis. Some key lessons in norovirus infection control were learned when the school doctor and other school staff members attempted to implement disease control measures. (1) Because vomiting aerosols and environmental contamination are likely sources of infection spread, thorough and systematic disinfection of schools, especially places where sick students are, should be carried out. (2) Staff should wear proper protective equipment, use effective disinfectants and follow correct procedures to handle vomitus. An alcohol-based hand sanitiser has a stronger ability to eliminate many common germs than soap, but may also contribute to the spread of illness; indeed, as the germicidal mechanism of alcohol is lipolysis, alcohol-based disinfectants are not effective in eliminating norovirus [45]. (3) The decision to suspend classes in a timely manner is paramount when dealing with norovirus outbreaks in schools, especially for person–person transmission.

There are several limitations that should be noted in this study. The student cases were children aged between 3 and 7 years old and were too young to participate in our case-control study; thus, the sample was relatively small.

In conclusion, the outbreak of norovirus in schools warrants the development of systematic training courses for school doctors and management staff. It may be necessary to ask the individuals in schools responsible for monitoring infectious disease to regularly review common infectious disease prevention and control strategies, with passing an examination being a requirement for employment. In general, outbreaks can be prevented if the vomitus and environment are properly and thoroughly disinfected by well-trained school personnel.

## Data Availability

The original data for this article can be found in Supplementary material.

## Supplementary material

For supplementary material accompanying this paper visit http://doi.org/10.1017/S0950268822000826. click here to view supplementary material
